# Digital Biomarker Representing Frailty Phenotypes: The Use of Machine Learning and Sensor-Based Sit-to-Stand Test

**DOI:** 10.3390/s21093258

**Published:** 2021-05-08

**Authors:** Catherine Park, Ramkinker Mishra, Amir Sharafkhaneh, Mon S. Bryant, Christina Nguyen, Ilse Torres, Aanand D. Naik, Bijan Najafi

**Affiliations:** 1 Interdisciplinary Consortium on Advanced Motion Performance (iCAMP), Michael E. DeBakey Department of Surgery, Baylor College of Medicine, Houston, TX 77030, USA; catherine.park@bcm.edu (C.P.); ram.mishra@bcm.edu (R.M.); 2 Telehealth Cardio-Pulmonary Rehabilitation Program, Medical Care Line, Michael E. DeBakey VA Medical Center, Houston, TX 77030, USA; amirs@bcm.edu (A.S.); msbryant@bcm.edu (M.S.B.); christina.nguyen@va.gov (C.N.); ilse.torresruiz@bcm.edu (I.T.); 3 VA Health Services Research and Development Center for Innovations in Quality, Effectiveness and Safety, Houston, TX 77021, USA; anaik@bcm.edu; 4 VA South Central Mental Illness Research, Education and Clinical Center (a Virtual Center), Houston, TX 77021, USA; 5 Department of Medicine, Baylor College of Medicine, Houston, TX 77030, USA

**Keywords:** physical frailty, frailty phenotype, machine learning, digital health, sit-to-stand test, wearable technology, older adults, remote assessment, telemedicine

## Abstract

Since conventional screening tools for assessing frailty phenotypes are resource intensive and unsuitable for routine application, efforts are underway to simplify and shorten the frailty screening protocol by using sensor-based technologies. This study explores whether machine learning combined with frailty modeling could determine the least sensor-derived features required to identify physical frailty and three key frailty phenotypes (slowness, weakness, and exhaustion). Older participants (n = 102, age = 76.54 ± 7.72 years) were fitted with five wearable sensors and completed a five times sit-to-stand test. Seventeen sensor-derived features were extracted and used for optimal feature selection based on a machine learning technique combined with frailty modeling. Mean of hip angular velocity range (indicator of slowness), mean of vertical power range (indicator of weakness), and coefficient of variation of vertical power range (indicator of exhaustion) were selected as the optimal features. A frailty model with the three optimal features had an area under the curve of 85.20%, a sensitivity of 82.70%, and a specificity of 71.09%. This study suggests that the three sensor-derived features could be used as digital biomarkers of physical frailty and phenotypes of slowness, weakness, and exhaustion. Our findings could facilitate future design of low-cost sensor-based technologies for remote physical frailty assessments via telemedicine.

## 1. Introduction

According to the World Health Organization, by 2050, approximately 22% of the global population will be 60 years or older [1]. Physical frailty, which is defined as the state of increased vulnerability in reserve and function across multiple physiological systems, is common in older adults [2]. The condition can increase the risk of adverse health outcomes, such as falls, poor quality of life, hospitalizations, mortality, etc. (see [3] for review). Although physical frailty is typically chronic and progressive in nature [4,5], it can be ameliorated or potentially reversed if identified and treated early [6,7,8]. Therefore, identification of older adults with physical frailty or at risk of becoming physical frailty plays an important role in monitoring health conditions, planning for appropriate health services, and designing and implementing interventions [9].

The medical profession generally relies on two common techniques to identify those at risk of physical frailty: a frailty phenotype and a frailty index. The Fried frailty phenotype assesses unintentional weight loss, slowness, weakness, exhaustion, and low physical activity [10]. A pre-frail/frail individual is identified when one or more of the five phenotypes are detected. The frailty index assesses health deficits (e.g., symptoms, signs, disabilities, diseases, etc.) [11]. The frailty index is represented as a ratio between the number of presented deficits and the number of considered deficits. However, the tools used for physical frailty assessment are resource intensive [12], and thus they are largely unsuitable for telehealth assessments and monitoring. Primary healthcare providers also need simpler tools to administer physical frailty assessments [13,14].

Wearable sensors, the internet of things (IoT), mobile technology, and cloud computing have encouraged medical device design engineers and researchers to use technology for frail-related developments [15]. We recently tested the effectiveness of five wearable sensors for a five times sit-to-stand test (5×STS) [16]; the sit-to-stand test is widely used in research and clinical practice to assess physical frailty and motor performance [17,18,19]. In our previous study [16], we found a strong correlation between sensor-based and manually-recorded 5×STS durations, and we first identified eight sensor-derived features as digital biomarkers for physical frailty and three key frailty phenotypes (slowness, weakness, and exhaustion). Despite these promising results, the use of five wearable sensors to extract eight sensor-derived features may have limited applications because of cost and computational burden.

Acknowledging this limitation, the present study explores whether a machine learning technique combined with frailty modeling could determine the least sensor-derived features needed for identifying physical frailty and three key frailty phenotypes (slowness, weakness, and exhaustion). We hypothesize that: (1) a machine learning technique combined with frailty modeling can determine the optimal features required for identifying physical frailty and three key frailty phenotypes (slowness, weakness, and exhaustion), and (2) fewer sensors can be used for determining the optimal features.

## 2. Materials and Methods

### 2.1. Participants and Experimental Protocols

This is a retrospective analysis of sensor data from 102 community dwelling older adults or Veterans, who participated in our previous work [16]. All participants were ambulatory volunteers aged 65 years or older, and they had no significant medical or psychiatric conditions and did not use assistive devices while standing and walking [16]. The study protocol was approved by the Institutional Review Board at the local institutional review boards including the Michael E. DeBakey Veterans Affairs Medical Center, Baylor College of Medicine, and the University of Arizona. All participants read and signed the informed consent form.

The Fried frailty phenotype assessed participants’ physical frailty from 0 to 5 based on five criteria (weight loss, weakness, slowness, exhaustion, and low physical activity) [10]. Based on the results, participants were classified into a robust group (RG, Fried frailty phenotype less than 1) or a pre-frail/frail group (FG, Fried frailty phenotype greater than or equal to 1).

Before performing the 5×STS, both groups were fitted with five wireless wearable sensors (LegSys+™, BioSensics, Watertown, MA, USA) [20] attached with Velcro to elastic belts worn on the trunk, left and right thighs, and left and right shanks. Each sensor had a tri-axial accelerometer and gyroscope, a Bluetooth module, a microcontroller, and a rechargeable battery. For the 5×STS, participants were instructed to sit on an ordinary chair, and fold their arms across their chest. After given the “go” instruction by a clinician, they performed the 5×STS as quickly as possible without resting their back or legs on the chair between the repetitions [21]. All participants completed the 5×STS successfully, and there were no system malfunctions during any of the experimental trials. Each sensor wirelessly transmitted quaternion data to the custom software installed on a standard laptop at a rate of 100 Hz.

### 2.2. Sensor Data Processing and Feature Extraction

Detailed information about the raw sensor signal processing and the determination of sensor-derived features for the three key frailty phenotypes (slowness, weakness, and exhaustion) are available in our previous work [16]. Briefly, sensor-based 5×STS duration and primary features were extracted from the five wearable sensors.

The eight primary features were hip angle range, hip angular velocity range, hip power range, knee angle range, knee angular velocity range, knee power range, vertical velocity range, and vertical power range. The eight primary features were computed for each STS cycle, and their mean and coefficient of variation (CV, defined as the standard deviation divided by the mean) were computed across 5×STS cycles. Therefore, the total number of sensor-derived features was 17 (i.e., sensor-based 5×STS duration + 8 primary features × 2 feature types (mean and CV)). Hip and knee angle, hip and knee angular velocity, and vertical velocity were computed by the raw sensor signal processing [16]. Angular power was computed from moment of inertia (I), angular velocity (ω), and angular acceleration (α) as:

(1) Angular power=I·α·ω =τ·ω

Hip and knee moment of inertia were estimated using adjusted Zatsiorsky-Seluyanov’s segment inertia parameters [22], and hip and knee angular acceleration were computed as the time derivative of hip and knee angular velocity. Vertical power was computed as a product of body mass, vertical velocity, and vertical acceleration. Vertical force was computed as a product of body mass and vertical acceleration. Scaled power considering weight and height was computed as:

(2) $$\mathrm{Scaled}\;\mathrm{vertial}\;\mathrm{power}=\frac{\mathrm{vertical}\;\mathrm{power}}{m\cdot g\cdot \sqrt{g\cdot h}}$$

where m is body mass, h is height, and g is gravitational acceleration. A scaled vertical power is unitless, and its computation is similar to the application of segment inertia parameters for calculating moment of inertia.

Consistent with our previous work [16], the indicators of slowness were the mean of hip angular velocity range, mean of knee angular velocity range, and mean of vertical velocity range; the indicators of weakness were the mean of hip angle range, mean of hip power range, mean of knee angle range, mean of knee power range, and mean of vertical power range; and the indicators of exhaustion were the CVs of the eight primary features.

### 2.3. Optimal Feature Selection and Evaluation of Frailty Modeling

To determine the features for optimal feature selection, either the one-way analysis of variance (ANOVA) or the Mann–Whitney U test was applied to the 17 sensor-derived features, depending on each sensor-derived feature’s normality as confirmed by the Shapiro–Wilk test. Eight of the 17 sensor-derived features showed a significant difference between the RG and FG, and thus they were used as independent variables for optimal feature selection.

Optimal feature selection used a recursive feature elimination technique with logistic regression modeling. The modeling used a frailty status (0 (robust) or 1 (frail)) as a dependent variable and the eight significant sensor-derived features as independent variables. The recursive feature elimination technique, which enables ranking the most effective features, was used to determine the least number of features that produce an optimal performance [23]. The bootstrapping technique, which enables testing any possible combinations of participants with different sample sizes, was used to generalize the logistic regression modeling [24,25]. Considering the number of participants (sample size; n = 102), the optimal feature selection used 2000 bootstrap iterations to optimize the robustness of logistic regression modeling, which is recommended in the literature [25].

**Figure 1:**
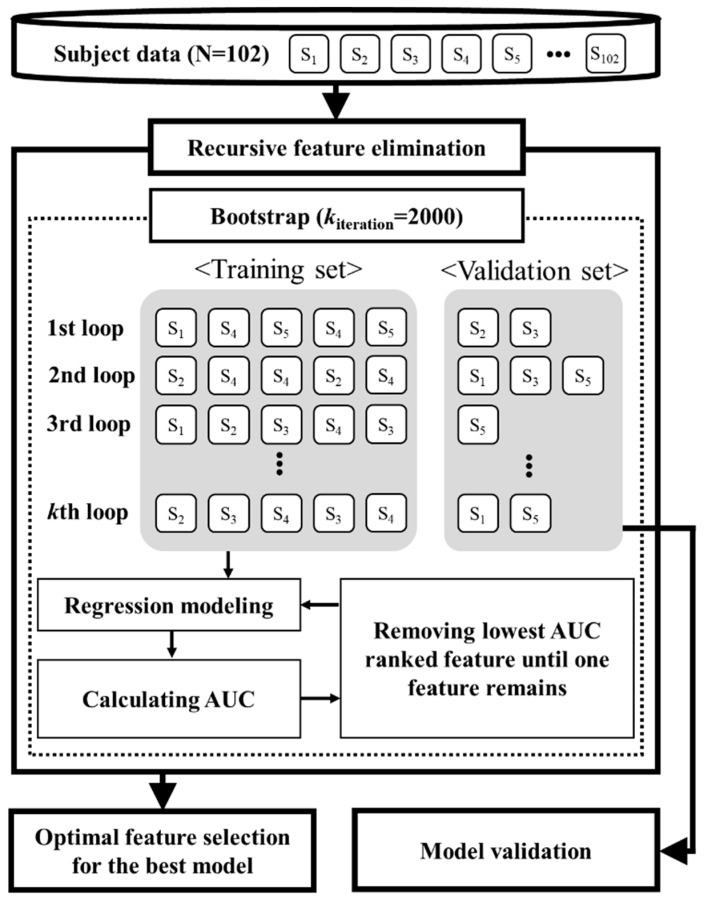
Flowchart of optimal feature selection and evaluation of frailty modeling.

Figure 1 shows the flow chart of optimal feature selection using the recursive feature elimination technique and the bootstrapping technique. The bootstrapping technique splits participants’ data into 2000 pairs of training and validation datasets, which enables calculating a 95% confidence interval (CI) for optimal feature selection. The five steps for recursive feature elimination are: (1) logistic regression models are created at each iteration loop. The number of logistic regression models is the number of significant sensor-derived features considered in each recursive loop (i.e., the first recursive loop considers the eight significant sensor-derived features, and they decrease by 1 after each recursive loop). For all logistic regression models, the dependent variable is the frailty status (i.e., 0 (robust) or 1 (frail)). For the nth logistic regression model, the independent variables are all significant sensor-derived features except for the nth feature; (2) for each model at each iteration loop, the receiver operating characteristic (ROC) curve and area under the ROC curve (AUC) are calculated because the AUC is a widely accepted performance measure to evaluate ranking predictions [26]; (3) AUC values across 2000 iterations are averaged for each model; (4) a sensor-derived feature with the lowest AUC value is removed; and (5) steps 1–4 repeat until only one sensor-derived feature remains (i.e., steps 1–4 correspond to one recursive loop, for a total execution of eight recursive loops is executed). Recursive feature elimination incorporating bootstrapping (i.e., 2000 pairs of resampling) runs 70,000 loops in total.

Model performance was evaluated by its AUC, sensitivity, specificity, and accuracy. Sensitivity and specificity are the ability of logistic regression models to identify participants with and without frailty, respectively. Accuracy is defined as:

(3) $$\mathrm{Accuracy}\;=\frac{\mathrm{TP}+\mathrm{TN}}{\mathrm{TP}+\mathrm{TN}+\mathrm{FP}+\mathrm{FN}}$$

where TP (true positive) and TN (true negative) represent the number of correctly identified frailty and the number of correctly identified non-frailty, respectively, and FP (false positive) and FN (false negative) represent the number of non-frailty identified incorrectly as frailty and the number of frailty identified incorrectly as non-frailty, respectively.

After determining the least optimal features, the performance of the logistic regression model with the least optimal features was evaluated. For AUC, sensitivity, specificity, and accuracy, mean and 95% CI were calculated from the validation datasets (i.e., 2000 iterations).

## 3. Results

Table 1 reports participants’ demographic characteristics for both groups, including statistical results. Statistical analysis found that weight and BMI were significantly higher for the FG than for the RG. However, age, gender, and height were not significantly different between the groups.

### 3.1. Significant Sensor-Derived Features

**Figure 2:**
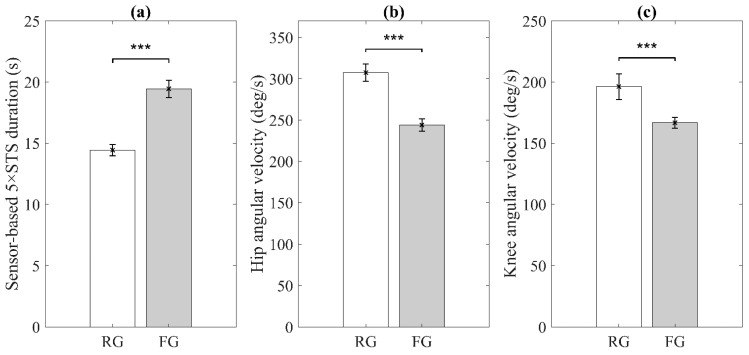
Significant sensor-derived features for slowness. (**a**) Sensor-based 5×STS duration; (**b**) Mean of hip angular velocity range; (**c**) Mean of knee angular velocity range. RG and FG denote robust group and pre-frail/frail group, respectively. Error bars indicate standard errors of the corresponding averages (*** *p* < 0.0001).

Statistical analysis showed that the eight sensor-derived features were significantly different between RG and FG. Figure 2 shows the results of the three sensor-derived features for indicators of slowness, including the statistical significance. Sensor-based 5×STS duration, mean of hip angular velocity range, and mean of knee angular velocity range were significantly slower for the FG than for the RG.

**Figure 3:**
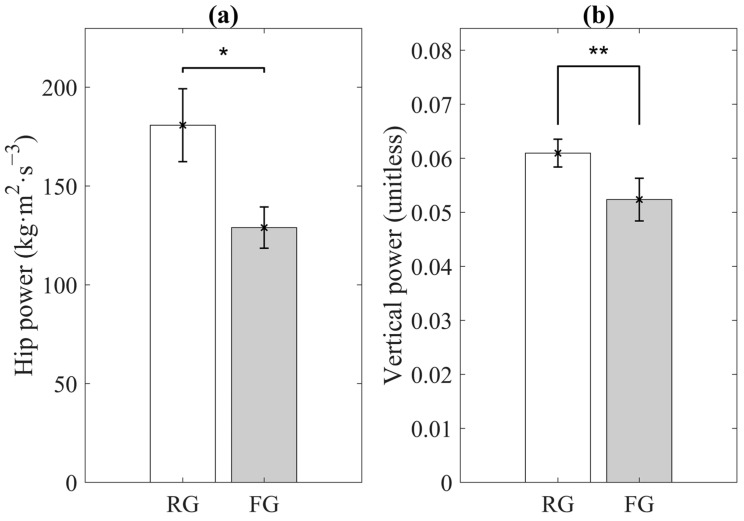
Significant sensor-derived features for weakness. (**a**) Mean of hip power range; (**b**) Mean of vertical power range. RG and FG denote robust group and pre-frail/frail group, respectively. Error bars indicate standard errors of the corresponding averages (* *p* < 0.05 and ** *p* < 0.01).

Figure 3 shows the results of the two sensor-derived features for indicators of weakness, including the statistical significance. Compared to the RG, mean of hip power range and mean of vertical power range were significantly lower for the FG.

**Figure 4:**
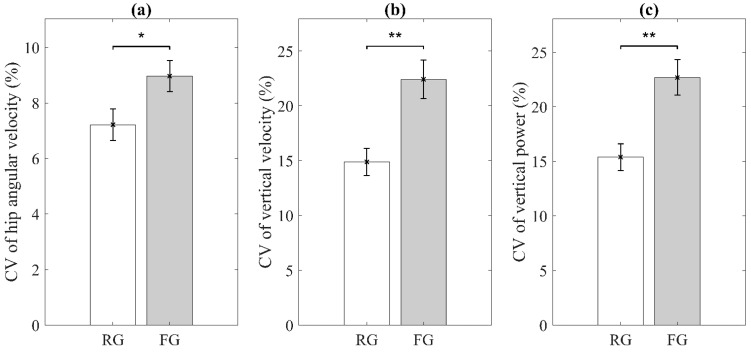
Significant sensor-derived features for exhaustion. (**a**) Coefficient of Variation (CV) of hip angular velocity range; (**b**) CV of vertical velocity range; (**c**) CV of vertical power range. RG and FG denote robust group and pre-frail/frail group, respectively. Error bars indicate standard errors of the corresponding averages (* *p* < 0.05 and ** *p* < 0.01).

Figure 4 shows the results of the three sensor-derived features for indicators of exhaustion, including the statistical significance. CV of hip angular velocity range, CV of vertical velocity range, and CV of vertical power range were significantly higher for the FG than for the RG.

### 3.2. Optimal Feature Selection and Evaluation

**Figure 5:**
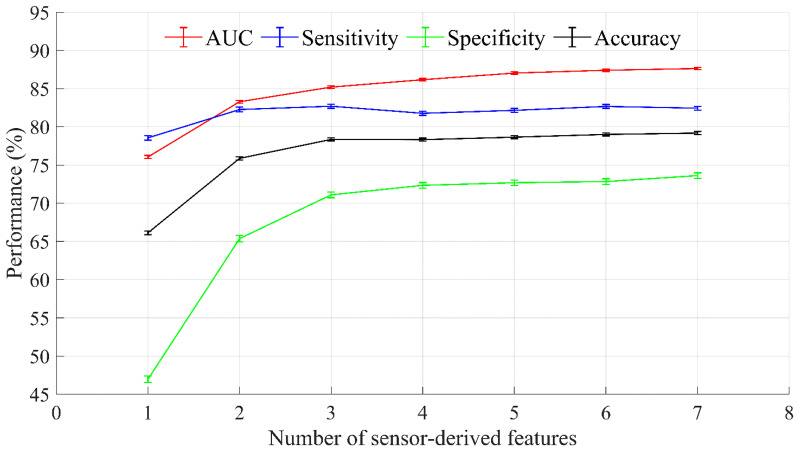
Results of optimal feature selection based on recursive feature elimination. Error bars indicate 95% confidence intervals.

Figure 5 shows the model performance assessed by AUC, sensitivity, specificity, and accuracy as a function of the number of ranked sensor-derived features based on the recursive feature elimination technique with logistic regression modeling. Table 2 reports the rankings of the eight significant sensor-derived features and an associated indication of the frailty phenotype. Based on the selection criteria (the presence of slowness, weakness, and exhaustion, and an AUC ˃ 0.8 (an AUC of 0.8 to 0.9 is considered excellent [27])), mean of hip angular velocity range, mean of vertical power range, and CV of vertical power range were selected as the optimal features. A logistic regression model with the selected features had an AUC of 85.20% (95% CI = 85.04‒85.36), a sensitivity of 82.70% (95% CI = 82.43‒82.96), a specificity of 71.09% (95% CI = 70.72‒71.46), and an accuracy of 78.35% (95% CI = 78.16‒78.54). The equation of a logistic regression model for the optimal features is:

(4) $$g\left(p\left(\mathrm{ph}\right)\right)=\ln\left(\frac{p\left(\mathrm{ph}\right)}{1-p\left(\mathrm{ph}\right)}\right)=\beta_{0}+\beta_{1}{\mathrm{ph}}_{1}+\beta_{2}{\mathrm{ph}}_{2}+\beta_{3}{\mathrm{ph}}_{3}$$

where g and ln is the logit function and the natural logarithm, respectively, p (ph) is the probability that the dependent variable equals the frailty status (i.e., robust or frail), and probability p (ph) ranges between 0 and 1. ph_1_, ph_2_, and ph_3_ indicate mean of hip angular velocity range, mean of vertical power range, and CV of vertical power range, respectively, and β_0_ is an intercept (2.722), and β_1_, β_2_, and β_3_ are constant coefficients (β_1_ = −0.022, β_1_ = 0.243, and β_3_ = 0.055), respectively. 

Table 3 reports the results of model validation. The mean values for an AUC, a sensitivity, a specificity, and an accuracy are 82.18%, 79.37%, 67.20%, and 73.91%, respectively.

## 4. Discussion

This study demonstrated the effects of the machine learning technique combined with frailty modeling (i.e., logistic regression modeling) for determining optimal sensor-derived features that is required to identify physical frailty and three frailty phenotypes (slowness, weakness, and exhaustion). The machine learning technique selected the mean of hip angular velocity range (indicator of slowness), mean of vertical power range (indicator of weakness), and CV of vertical power range (indicator of exhaustion) as the optimal sensor-derived features. The performance of the machine learning technique showed excellent AUC (85.20%) and high sensitivity (82.70%), specificity (71.09%), and accuracy (78.35%). Different from the published literature [28,29,30], this study first showed that the FG had a slower, weaker, and more exhausted performance of the 5×STS compared to the RG.

Physical frailty is reversible when identified and treated early (see [7] for review), and a routine and accurate assessment of physical frailty is a crucial part of intervention and treatment [2,10]. Although a variety of physical frailty assessment tools exist [12,13], the Fried frailty phenotype and frailty index are widely used for in-person assessment and monitoring. They are inadequate, however, for remote assessments via telemedicine. For example, the frailty phenotype requires equipment for weakness assessments (e.g., handgrip dynamometer) and enough physical space for slowness assessments (e.g., 4.57 m walking test) [5], and the frailty index relies on patient-reported outcomes that are relatively subjective compared to the frailty phenotype [6]. Additionally, trained health professionals must administer assessments in person and interpret the results. Given poor compliance, increased medical costs, and stress on family or caregivers, sensor-based physical frailty assessment tools offer a simple, fast, and objective physical frailty assessment protocol irrespective of physical setting.

Our results indicate that the frailty model with three optimal features (i.e., mean of hip angular velocity range, mean of vertical power range, and CV of vertical power range) had a lower AUC of 85.20% (95% CI = 85.04‒85.36), a specificity of 71.09% (95% CI = 70.72‒71.46), and an accuracy of 78.35% (95% CI = 78.16‒78.54) compared to an AUC (87.64% (95% CI = 87.49‒87.79)), specificity of 73.61% (95% CI = 73.25‒73.97), and accuracy (79.18% (95% CI = 78.98‒79.39)) of the frailty model with eight sensor-derived features, as shown in Figure 5. However, both models showed an excellent AUC and a high specificity and accuracy [23,27] and had a similar sensitivity levels (the model with three optimal features: 82.70% (95% CI = 82.43‒82.96) and the model with eight sensor-derived features: 82.44% (95% CI = 82.19‒82.69)). Notably, the three optimal features are sufficient to identify physical frailty and the three key frailty phenotypes, and two wearable sensors (trunk and one thigh) can capture the three optimal features. Compared to the use of a five-sensor configuration, a two-sensor configuration has significant commercial advantages (e.g., cheaper to manufacture and simpler to integrate), and the health care advantages include easier use and minimal computation when analyzing and interpreting the results.

Compared to other function tests (e.g., walking and strength tests), the 5×STS is simple, fast, safe, easily reproducible, and widely used in research and clinical practice [17,18,19,31]. The 5×STS is an important component of the Short Physical Performance Battery, a clinical tool used for identifying physical frailty [32]. Therefore, sensor-based 5×STS could enable health professionals to quickly identify slowness, weakness, and exhaustion, which can assist in recognizing potential modifiable risk factors to improve health outcomes and strategies for older adults [33,34].

The limitations of this study include: the possible mispredictions of physical frailty and three frailty phenotypes, and a possible ungeneralizable frailty model. We speculate that the possible mispredictions of physical frailty and three frailty phenotypes are due to the differences between the 5×STS and the Fried frailty phenotype method. For example, slowness of gait may not be indicated by slowly performing 5×STS, and weakness of grip strength may not be indicated by weakly performing 5×STS. Our speculation is also supported by our previous findings that a rapid sensor-based elbow flexion/extension test with a machine learning approach identified physical frailty and frailty phenotypes diagnosed with the frailty index with an accuracy of above 80%, and possible mispredictions with less than a 20% false rate [23]. In addition, we attribute the possible mispredictions of physical frailty and three frailty phenotypes to the binarization of the Fried frailty phenotypes. Future research will focus on improving prediction rates by including additional measurements with sensors (e.g., gait speed) and by using a multiclass classification method. Although we have used the bootstrapping technique to generalize logistic regression modeling, our current sample size (n = 102) and gender imbalance may not be sufficient, given our cross-comparisons and combinations of participants. Therefore, future research will use larger samples with balanced gender within and between groups. We also plan to use a telemedicine camera (i.e., laptop or tablet integrated camera), which we are currently developing to record older adults performing 5×STS without wearing sensors. Our aim is to demonstrate that sensorless 5×STS could be an alternative when there is limited or no access to sensors.

## 5. Conclusions

This study demonstrated that the mean of hip angular velocity range, mean of vertical power range, and CV of vertical power range are the optimal features for identifying physical frailty and three key frailty phenotypes (slowness, weakness, and exhaustion) with sensor-based 5×STS in older adults. Of note, the three optimal features can be extracted with the two-sensor configuration, which could facilitate the technological development and commercial application of low-cost, easy-to-use, computationally efficient sensor-based frailty assessment tools. 

Reliance on telemedicine is expected to increase in the post-COVID era [35]. Our sensor-based or sensor-less 5×STS would enable remote physical frailty assessments of older adults performing the 5×STS at home. Older adults and their families and caregivers should also benefit from remote physical frailty assessments via telemedicine.

## Figures and Tables

**Table 1:** Demographic data for robust group (RG) and pre-frail/frail group (FG).

	No./Total No. (%) by Group		*p*-Value
	RG (n = 42)	FG (n = 60)	
Age, years	74.79 ± 6.64	76.57 ± 8.00	0.085
Female, n (%)	34/42 (81.0)	39/60 (65.0)	0.079
Height, cm	162.09 ± 7.34	164.90 ± 10.77	0.230
Weight, kg	66.77 ± 12.21	78.61 ± 19.95	0.001 *
BMI, kg/m^2^	25.40 ± 4.23	28.70 ± 5.79	<0.0001 *

Values are presented as mean ± standard deviation (SD) or n (%). Asterisks denote the significant difference between groups.

**Table 2:** Ranking sensor-driven features.

Rank	Sensor-Driven Features	Phenotype	Sensor Configuration
1	Mean of hip angular velocity range	Slowness	Trunk/Thigh
2	Mean of vertical power range	Weakness	Trunk
3	Coefficient of Variation (CV) of vertical power range	Exhaustion	Trunk
4	CV of vertical velocity range	Exhaustion	Trunk
5	Mean of hip power range	Weakness	Trunk/Thigh
6	Sensor-based 5×STS duration	Slowness	Trunk/Thigh/Shank
7	Mean of knee angular velocity range	Slowness	Thigh/Shank
8	CV of hip angular velocity range	Exhaustion	Trunk/Thigh

**Table 3:** Model validation.

Validation Metric	Mean	95% Confidence Interval
AUC (%)	82.18	81.93 to 82.43
Sensitivity (%)	79.37	78.92 to 79.84
Specificity (%)	67.20	66.64 to 67.76
Accuracy (%)	73.91	73.63 to 74.19

AUC: area under the receiver operating characteristic curve.

## Data Availability

The datasets are available upon request to the corresponding author.
